# Emergence versus Reductionism in Science Publications

**DOI:** 10.1177/00221465251335041

**Published:** 2025-05-23

**Authors:** Troy Duster

**Affiliations:** 1 University of California, Berkeley, CA, USA

**Keywords:** DNA, diabetes, emergence, genetics, race, reductionism

## Abstract

Just a few years after the U.S. government’s decision to fully fund the Human Genome Project (HGP) in 1990, an important harbinger of things to come was the publication of the controversial 1994 book *The Bell Curve* by Richard J. Herrnstein and Charles Murray. The authors’ most controversial claim was that human intelligence was at least 60 percent genetic. At that time, the national advisory group to the HGP, the Ethical Legal and Social Implications committee (ELSI) requested that the *American Journal of Human Genetics* critique and respond to the authors’ claim. The editorial board of the journal refused on the grounds that “this book was about behavioral genetics” while the HGP was about human molecular genetics. Members of ELSI committee argued vigorously that this distinction between different forums and platforms used to explain human genetic variation would soon collapse and merge. However, it was only a matter of time before behavioral geneticists would claim the legitimacy of being under the mantle of molecular genetics. In this address, I show just how prescient the ELSI group had been. Much of the answer lies in the reward structure for science publications that strongly favor reductionism versus emergence.

At the dawn of the Human Genome Project in the late 1980s, Nobel Laureate Walter Gilbert gave lectures in which he would famously pull a compact disc (CD) out of his pocket, hold it up to the audience, and say: “Twenty-years from now, you will be able to hold up a CD like this and say, ‘Here is a human being, it’s me!’” Indeed, Gilbert’s (1992) essay, notably entitled “A Vision of the Grail,” suggested that once the mapping and sequencing has been completed, the human project to “know who we are” will be nearly complete. To recognize that we are determined by a finite collection of information that is knowable, he said, will change our view of ourselves: “It is the closing of an intellectual frontier, with which we will have to come to terms” (Gilbert 1992:96).

Although Gilbert was indulging a large dose of hyperbole, elements of the claim remain and for a reason. The idea that we are we really just the sum of the small component parts that constitute our deoxyribonucleic acid (DNA) makeup is both too simple to be true but apparently too seductive to dismiss. Indeed, a 2023 interview with Jennifer Doudna, winner of a Nobel Prize for her work on CRISPR (clustered regularly interspaced short palindromic repeats), has echoes from the early days of the Human Genome Project (HGP). The article is titled “Suddenly, It Looks Like We’re in a Golden Age for Medicine” (Wallace-Wells 2023), and Doudna suggests that in the very near future, the new technological developments with immune therapies will likely cure a host of diseases and health challenges that have so far proved elusive to medical treatments.

Feedback loops, interaction effects, and an ever-reengaging dialogue between the genes and the environment (physical and social, interior and exterior—even epigenetics) have made the reference to Gilbert’s (1992) compact disc a deeply flawed metaphor. Yet the attraction to the idea is powerfully seductive and has been for much of human history. Long before the science of genetics, humans have conjured a hereditary determinism to explain their fates.

In those early years of the mapping, Leroy Hood, a premier molecular biologist and a primary advocate of the HGP, also championed the potential health-giving prospects of the HGP. He speculated that once the project is completed, there would be extraordinary potential for understanding genetic diseases and a host of other human behaviors with a genetic component. He argued that the most important area of DNA diagnostics would be the identification of genes that predispose individuals to disease. He acknowledged that many such diseases—cardiovascular, neurological, and autoimmune—are polygenic, but what he meant was that they are the result of the action of two or more genes (Hood 1992:155).

Thus, even while acknowledging a polygenic and interactional feature, Hood’s (1992) formulation actually focuses the analysis to the interaction of the genes. As noted, Gilbert and Hood, both preeminent molecular biological scientists, each made a substantial contribution to the theoretical underpinnings of the HGP. Their contrasting views of the ultimate meaning and status of the HGP sharply delineate a major thesis to be developed here, a resurgent conflict in the scientific community about the importance of emergence versus the importance of reductionism. Just a few years after the U.S. government’s decision to fully fund the HGP, an important harbinger of things to come was the publication of *The Bell Curve* (Herrnstein and Murray 1994). The most controversial claim of the authors was that human intelligence was at least 60 percent genetic (Herrnstein and Murray 1994). At the time, the national advisory group to the HGP, the Ethical Legal and Social Implications committee (ELSI), requested that the *American Journal of Human Genetics* critique and respond to this claim. The editors of the journal refused on the grounds that “this book was about behavioral genetics” while the HGP was about human molecular genetics. Members of ELSI committee argued vigorously that this distinction between different forums and platforms used to explain human genetic variation would soon collapse and merge. We argued that it was only a matter of time before behavioral geneticists would claim the legitimacy of being under the mantle of molecular genetics. As I elaborate in this article, it will be clear just how prescient the ELSI group had been.

## Emergence versus Reductionism—and Human Genetics

In the realm of scientific discourse about human attributes and human behavior, there are now and always have been two sharply competing versions of how to best account for a full range of phenotypical expressions, clinical manifestations of disorders and diseases, and even such complex aspects of human existence as the functioning of a powerful and effective immune system. This includes a dizzying array of behaviors, from alcoholism to same-sex preference, from intelligence and scholastic achievement to crime.

A reductionist strategy attempts to explain the phenomenon under investigation in terms of a unit interpreted as the most essential to explain that phenomenon. The unit presumed to be the more essential is likely to be the more powerful predictor, and thus for a number of reasons to be elaborated in the following, the mantle of the “more scientific” moves in the direction of the more reductionist claim. Even though scientists typically acknowledge the limits of reductionist approaches, there are structural reasons in the organization and production of scientific knowledge that help explain why an emphasis on reductionist strategies has dominated much of scientific inquiry into human behavior for the last several decades.

In sharp contrast to reductionism is the idea of emergence. Every student who has encountered chemistry (and vials of chemicals) knows that “emergence” is a key element in both scientific thought and human experience. Emergence, quite simply, is the idea that the phenomenon to be explained emerges out of the interplay of the component elements and that, most importantly, the phenomenon cannot be explained by reference to an analysis of the component parts that separated from each other. An obvious example is an explosion. Nothing much of interest occurs when a vial of nitrogen is a few inches away from a vial of glycerin. But placed together, a phenomenon emerges that cannot be explained with assumptions about locating “a cause” in either of these two single isolated constituent elements—an explosion. Or even more prosaically, there is water, which is not “caused” by either hydrogen or oxygen. Philip Anderson, who won a Nobel Prize in 1977 for his work in condensed matter physics, wrote what is widely regarded as the most persuasive account of emergence that appears in the scientific literature:

A water molecule is simple enough, with two small hydrogen atoms attached to one large oxygen atom. The now well understood principles of atomic physics govern the behavior of this molecule. However, when “billions upon billions” of those molecules are in the same pot: Suddenly you’ve got a substance that . . . has collectively acquired a property, liquidity, that none of them possesses alone . . . there’s nothing in those well understood equations of atomic physics that even hints of such a property. The liquidity is “emergent.” (Waldrop 1992:82).

And just as liquidity is emergent (i.e., not to be explained by its elementary constitutive parts), so too is water vapor. Heating the liquid produces a phase transition into vapor, and the spiral goes upward: Take your water vapor out over the Gulf of Mexico, and let it interact with the sunlight and wind, and it can organize itself into an emergent structure known as a hurricane. Anderson (1972) went on to point out that “life itself is an emergent property,” as is the mind, the product of billions of neurons obeying the biological laws of living cells. And as he notes in that article: “At each level of complexity, entirely new properties appear. [And] at each stage, entirely new laws, concepts, and generalizations are necessary, inspiring inspiration and creativity to just as great a degree as in the previous one. Psychology is not applied biology, nor is biology applied chemistry” (Anderson 1972:393).

But why go on about this emergent–reduction distinction? Almost everyone genuflects to the idea that emergent strategies of explanation are legitimate in scientific inquiry. Even the most ardent reductionists give lip service to the idea that of course, there is interaction among elements, and almost everyone genuflects to the idea that emergent strategies are legitimate in scientific inquiry and theorizing. Indeed, perhaps the most famous and cited quote in Western scientific medicine is Hippocrates’s essay “On Airs, Waters, Places,” a quintessential statement of emergence and interaction. It survives not only for its historical significance, written more than 2,000 years ago, but it also is a compelling account.

Contemporary epidemiology has a way of discussing emergence through a particular lens, called the “web of causation.” If no single factor explains the occurrence of a disorder, and scientists quickly acknowledge this to be the case, then we can see the “window of opportunity” for a wide range of parallel and sometimes counter-scientific accounts, from folk wisdoms to theology, from philosophical ruminations about the location of blame and culpability to the circuitous route of justice and retribution, from diet and nutrition to lifestyles and worldviews.

Even during the plague of the middle of the fourteenth century, not everyone who was exposed to the high levels of contagion was afflicted. The plague wiped out a quarter of the population, and for the next three centuries, it was the scourge of Europe. Nonetheless, in the eighteenth century, it not only greatly subsided but largely disappeared . . . long before effective focused understanding and treatments were available.

During the nineteenth century, tuberculosis was common in select areas of the globe, but again, not everyone who was exposed to the tubercula bacillus succumbs. The same was true for cholera and yellow fever. Clearly, there is a complex, multifactorial interaction of factors that explains most illnesses. A single causal factor is rarely unconditionally determinant. Again, although scientists will almost universally agree with this truism, the attraction toward the opposite pole of reductionism in twentieth-century science has been an ineluctable force, a powerful magnet pulling research, specialization, the division of labor, and the structure of rewards to gravitate in orbit around discoveries of “magic bullets.”

Why, then, has the “science” bias been toward the isolation of a particular, single force? In 1989, it was announced that there had been the discovery of the gene for cystic fibrosis. However, upon closer inspection, two things were true about this development that would escape the attention of the ordinary reader. First, there are many mutations of the cystic fibrosis gene, and the “discovery” of a particular gene, or marker for that gene, does not explain the full range of what constitutes the clinical manifestation of cystic fibrosis. Second, and even more important, even when one has specified that particular gene, the way in which the gene expresses itself is so variable that some with “the exact same genetic diagnosis or designation” can live for many decades, others are unable to survive childhood, and others with the same diagnosis can have major life-threatening or relatively minor health problems. In other words, because cystic fibrosis is not one phenomenon that expresses itself phenotypically in one manner, there is necessarily a large role for “emergent,” “complex,” or “interactive” forces and elements to contribute to an explanation.

There are examples of important successes with a reductionist strategy. For example, for decades, stomach ulcers were thought to be primarily a function of one’s diet. The medical profession routinely suggested a shift to mild versus spicy foods. Then with the discovery of the bacteria called *Helicobacter pylori* (*H. pylori*), our whole understanding of ulcers was completely upended. It is now believed that most ulcers are caused by *H. pylori*, and new treatments for ulcers were designed with this new understanding.

## Incentive structures and publication pressures

Much like pressures on corporate executives to produce positive results for quarterly and annual reports, the academy now places measurable pressures on faculty to produce annual reports heavily focused on their publications. Many colleges and universities have “point systems” that literally assign points for the number and placements of publications cited on one’s curriculum vita. In such an environment, researchers and scholars attempting to explain “emergent phenomena” (i.e., outcomes of the interplay and interaction of forces that cannot be reduced to a single explanatory variable) are at a considerable disadvantage. Let’s take educational achievement as an example. A student’s educational success is a consequence of a complex interplay of familial, social, political, economic, and cultural forces. Nonetheless, a publication claims that 74 points in the genomic structure explain some “advantage.” In this study, with a sample of 293,723 individuals, the empirical referent for educational success was a few more years of schooling and claimed: “Because educational attainment is measured in large numbers of individual, it will continue to be useful as a proxy phenotype in efforts to characterize the genetic influences of related phenotypes, including cognition and neuropsychiatric diseases” (Okbay et al. 2016:539; this article listed more than 80 coauthors in its online version).

In sharp contrast, consider that these three classic social science studies woven together constitute an emergent explanation that advances our understanding like the successful patching together of pieces in a jigsaw puzzle.

First, a classic historical example of a researcher trying to explain the emergent features of educational achievement, from (1) teacher expectations (*Pygmalion in the Classroom*, Rosenthal and Jacobson 1968) to (2) counselor guidance (*Educational Decision Makers*, Cicourel and Kitsuse 1963) to (3) Willis’s (1977) classic study of how working-class students in Birmingham regarded as class traitors those among them who tried to meet middle-class teachers’ expectations (*Learning to Labour*). These elements of teacher expectations documented in *Pygmalion in the Classroom* dovetail and emerge to explain so much more of the variance than the reductionist “74 loci in the genetic structure,” but it requires several years to complete such a study. Each of these three studies required a full monograph, impossible to collapse into a journal article. Yet fields of inquiry characterized as “the more scientific disciplines” tend to devalue book manuscripts and routinely favor peer-reviewed articles in the discipline’s highest ranked journals.

An even more striking example comes from the study of diabetes and explanations in the literature about its causes. Before turning to that topic, some backgrounding is needed to set the context. In December 2007, *Science* named genome-wide association studies as the major “breakthrough of the year” in the study of human molecular genetics (Pennisi 2007). Within the next two years, researchers published articles reporting these “associations” with 80 diseases and traits (Hindorff et al. 2009). The new development was a technique that permitted science researchers to place tens of thousands of genetic material from different human populations to search for patterns or associations of those patterns with health and social outcomes. This statistical approach is an exploratory strategy that researchers consider to be “hypothesis-generating” rather than “hypothesis-driven.” (Fujimura and Rajagopalan 2011).

### Comparing a Reductionist Strategy to an Emergent Path to Explain Diabetes

Now we turn to the main empirical case to illustrate the sharp, even dramatic difference between (a) a completely ahistorical reductionist strategy to explain the surging rate of type two diabetes in the last three decades—and contrast it with (b) an emergent approach that takes into account interlocking interactive elements across a full century.

“There is no race which is so subject to diabetes as the Jews,” wrote W. H. Thomas in 1904. Thomas, a New York physician was voicing an almost universally held belief in the United States that of all the “races,” Jewish people had the greatest likelihood of developing diabetes. At the same time, most members of the medical community considered the prevalence of diabetes among Black individuals to be unusually low. In the words of a Johns Hopkins physician in 1898, “Diabetes is a rare disease in the colored race” (Tuchman 2011:24).

A century later, things have changed dramatically. Jewish persons are now routinely categorized together with White Americans of European descent—and White persons have less than half the rate of diabetes relative to African American persons. The link between Jewish origin and diabetes dates back to the European medical literature, most particularly, in the late nineteenth-century writings of Joseph Seegen of Vienna. Tuchman (2011:25) reports that “After Seegen noted in 1870 that roughly one quarter of his 140 diabetes patients were Jewish, other studies started appearing alleging that Jews died of diabetes at a rate between two and six times higher than the rest of the population.” In the German literature, diabetes even came to be known as the *Judenkrankheit* or “Jewish disease.”

When J. G. Wilson, a physician with the U. S. Public Health Service, tried to understand why the diabetes mortality rate in New York City had tripled between 1889 and 1910, he compared the rapid growth in the city’s Jewish population with the rise in the diabetes mortality rate. For Wilson, the correlation between these two sets of data was sufficient to demonstrate causation. To explain why Jewish people experienced such a high rate of diabetes, Wilson turned to racial traits, claiming that “some hereditary defect” made Jewish people more prone to develop the disease. He did not elaborate on the nature of the “defect,” but others pointed to the supposedly sensitive nervous system of the Jewish people. For Osler, it was the Jewish individuals’ particularly “neurotic temperament.” For the author of an article in the widely read *Collier’s Magazine*, it was the Jewish people’s “racial tendency to corpulence” (Tuchman 2011:25).

Although the Pima Indians of Arizona have long since replaced Jewish people as the group with the highest reported risk of diabetes, the method of recording a snapshot of corpulence (now cast as body mass index) continues the tradition of collecting cross-sectional data on the physical characteristics of the target population.

The question of how best to approach a strategy to increase the health of disenfranchised and economically distressed persons is a hotly contested issue, mainly because of the overlap of poverty, illness, ethnicity, and race (Keller et al. 2012). This overlap has led some to the conclusion that there is something basically different in the biogenetic makeup of different groups that might best explain health disparities. The Pima Indians have the highest rate of diabetes of any population ever studied, and they have become the subject of intense scrutiny and research as to why. “More than half of the Pima older than 35 years of age have the disease, and the prevalence rates reach a peak of 86 percent in women aged 55 to 64” (Johnson, Nowatzki, and Coons 1996:97). The prevalence rate increased by 42 percent in the decade between 1967 and 1977 (Carter et al. 1989). Here is an excerpt from an account of the approach supported by the National Institutes of Health (NIH) that I have termed elsewhere “looking inside the body” for answers.

Beginning in 1983 and continuing for 10 years, the National Institute of Diabetes and Digestive and Kidney Diseases (NIDDK) studied the genetic codes of almost 300 nondiabetic Pima Indians. After a number of years, some of the volunteers developed diabetes and Dr. Clifton Bogardus, III who served as chief of the Clinical Diabetes and Nutrition section of NIDDK from 1985 to 2000, and his team were able to determine that insulin resistance and obesity were major predictors of disease (Lillioja et al. 1993).

Because diabetes is such a complex disease, Dr. Bogardus and his staff were attempting to narrow their search by first looking for the genetic causes of physical conditions that can lead to diabetes, such as the genes that influence a person’s cells to secrete less and respond less to the insulin that is needed to regulate blood sugar. In 1993, they identified a gene called FABP2 that may contribute to insulin resistance. This gene makes an intestinal fatty acid binding protein using one of two amino acids. When the gene makes the protein with threonine, one of those amino acids, the body seems to absorb more fatty acids from the fat in meals. NIH scientists think that could lead to a higher level of certain fats and fatty acids in the blood, which could contribute to insulin resistance (Mitchell et al. 1995).

On the matter of potential known effective interventions, this approach does not have (indeed, could not have) much of a track record. For at least the last half century, we have known that the rate of type two diabetes among Native Americans is more than double that of White individuals in the United States. Until quite recently, the dominant theory among geneticists who have approached this topic has strongly suggested that Native Americans are far more likely to possess genes that enable fat-hoarding, sometimes labeled “thrifty genes.” They hypothesized that these genes were conducive to adaptation because the ancestors had a need to survive during cycles of famine. However, because this group now lives in a world in which they can routinely ingest foods with high fat and sugar content, these putatively formerly protective/adaptive genes are now placing this population at greater risk for diabetes.

A 2012 study suggests that it was the high-fiber diet that “locked in to place” the thrifty genes, not the adaptive mechanisms generated by famine cycles (Reinhard et al. 2012). Notice that in both accounts, genes are playing a dominant role in explaining “health disparities.” Yet we have strong evidence that diet has far greater analytic explanatory power, across all groups, when addressing the diabetes crisis that has struck across the globe since 1980. The fastest rate of increase is in India, which the World Health Organization has called the diabetes capital of world. Current estimates suggest that at least 35 million suffer now, and best estimates predict this figure will double in the next decade (Siegel, Narayan, and Kinra 2008). In the last three decades, the growing middle classes of India have experienced a dramatic increase in rates of diabetes, and “thrifty genes” have less to do with this than the new capacity of the newly well-to-do to consume high levels of sugar in the many ceremonies and ritual dinner celebrations that they can now afford.

To return to the extraordinarily high rate of diabetes among the Pima, an alternative to the genetic approach sets the analytic frame in a broader socio-historical context. Those who approach the matter from this angle have a very different view of how to think about diabetes prevention and treatment. In Table 1 on “prevalence of diabetes in related populations,” note the striking pattern of urban versus rural dwelling among six populations across the globe. Those who live in urban areas and consume a westernized diet have a very high rate of diabetes, but those who have lived in “traditional” sites where they practice “traditional culture” hardly experience any diabetes:

**Table 1.** Prevalence of Diabetes in Related Populations.

	Traditional	Prevalence (%)	Westernized	Prevalence (%)
Amerindians	Mapuche	0	Pima	23
New Guinea	Rural	0	Urban	37
Australian Aborigines	Rural	0	Urban	23
Middle East	Yemen	4	Lebanon	14
Chinese	Rural China	0	Urban Taiwan	13
Asian Indian	Rural India	0	Fiji	22

*Source:* King and Rewers (1993).

Of course, what is most striking about Table 1 is that this pattern holds true for every group sampled, across a wide swath of the globe. Did they all have thrifty genes, and if so, what intervention is implied other than a dramatic shift in diet? Or from another perspective, since the sharp increase in type two diabetes has come about in the last three decades—just in pure scientific logic, far more of the variance is explained by a systematic empirical investigation of shifting patterns of nutritional intake.

In a summary of the problems encountered when trying to explain sharp rates of difference between the tribes studied, Carter et al. (1989:) concluded:

We have no data on relative rates of obesity or lifestyle differences that might explain the different rates of diabetes between the Pueblo tribes and the tribes of the Athabascan groups. . . . Whether tribes of the Athabascan language group carry a genetic risk of diabetes different from that of the Pima and Pueblo Indians is unknown.

A few years after the Carter et al. (1989) article was published, a study was undertaken to focus on lifestyle as a potential explanatory force. Pima Indians using an outpatient hospital pharmacy in southern Arizona were invited to participate (Johnson et al. 1996). Subjects were given a self-administered questionnaire that probed for demographic information and clinically relevant variables and then were asked to take a short-form version of a health survey. Notice the inverted parallel to the situation described in the introduction—where data collected on patients’ heart condition was confined to the doctor’s office. But although many of the heart patients assessed at routine checkups were deemed otherwise healthy, the Pima participants included in the study were restricted to those known to have diabetes. And although the title of the study had in it the name “lifestyle,” the only data collected were within the confines of a medical establishment, a hospital pharmacy visit.

These are “snapshots” or “freeze-frame” accounts of the condition of a population at a single point in time. What happens when we step back and try to situate the Pima Indians’ health crisis around diabetes in a larger sociocultural context, situating this group outside the hospital and investigating instead the “natural setting” in which their lives have been shaped. A good ethnography begins with a sociocultural history of the group being studied, and we can learn much that has direct relevance to their current high rates of diabetes from just a brief overview of that history.

At the end of the nineteenth century, the Pima were known to be superb farmers, self-sustaining and independent (DeJong 2007:59). They had lived for centuries near the free-flowing Gila River, which supplied ample water for their agricultural needs. Indeed, they called themselves “Akimel O’tham,” or “River People.” However, expansion of White settlers westward would dramatically change that lifestyle and force them to abandon water-intensive crops. By the turn of the twentieth century, thousands of these White settlers lived next to the Pima Reservation and began competing directly for irrigation rights. As early as 1877, the Desert Land Act required that an applicant required bona fide application of water to the land to obtain title. Ultimately, dams were built that redirected water away from traditional Pima farms, forcing residents to either abandon the area or shift to a different source of livelihood (DeJong 2007:48). In 1902, a health survey found only a single case of diabetes among the Pima. Three decades later, the number had increased to more than 500. Then, during the 1930s, the Coolidge Dam was completed. Although it was heavily touted to bring water to all, within a few years, it became clear that the Pima were not to be the beneficiaries. There was certainly not enough water directed their way to restore traditional farming. Poverty was taking a heavy toll, with early deaths rising precipitously among the Pima, and the federal government embarked on a program to provide free government surplus food to the community.

Thus begins the substantive and compelling account of the dramatic increase of diabetes in this population. It was free food, but it was saturated with a diabetic person’s nightmare: refined white flour, processed cheese, lard, candy and chips, refined sugar, grape juice, and lots of macaroni. Anthropologists monitoring the dietary circumstances noted that the diet of the Pima from earlier times consisted of wild plants and game animals, when they used such foods as seeds, buds, fruits, and joints of various cacti; seeds of the mesquite, ironwood, palo verde, amaranth, salt bush, lambsquarter, horsebean, and squash; acorns and other wild nuts; and “roots and bulbs of the sandroot (wild potato) . . . deer, antelope, . . . rabbits, quail, dove, wild ducks, wild turkey” (Mark 1960:46).

Fast forward to the middle of the twentieth century, when this diet was completely obliterated (Hackenberg 1962), and in its place, the Pima received boxes piled on boxes of processed macaroni and cheese, where the larger the family size, the more entitlement to those free boxes of food. In the 1890s, the dietary intake of fat was 15 percent, but by the 1990s, it had nearly tripled to an eye-popping 40 percent (NIDDKD 1996:19).

In sum, whether attempting to explain high rates of diabetes in a population or “educational attainment as measured by number of years in school,” in recent decades of science funding, the reward structures bend increasingly toward reductionism and away from emergence.
